# Atrial Fibrillation Ablation Across an Inferior Vena Cava Filter

**DOI:** 10.7759/cureus.109739

**Published:** 2026-05-27

**Authors:** Wassim Beladel, Amine El Houari, Karim Hasni, Oussama Cheikhna, Mohamed El Minaoui

**Affiliations:** 1 Department of Cardiology, Souss-Massa University Hospital, Faculty of Medicine and Pharmacy of Agadir, Ibn Zohr University, Agadir, MAR; 2 Department of Cardiology, Toulon Hospital Center, Toulon, FRA

**Keywords:** atrial fibrillation, atrial fibrillation ablation, cardiac electrophysiology, inferior vena cava filters, radiofrequency ablation (rfa)

## Abstract

Catheter ablation with pulmonary vein isolation is a well-established treatment for symptomatic atrial fibrillation, particularly in patients intolerant to antiarrhythmic therapy. However, the presence of an inferior vena cava (IVC) filter represents a technical challenge for standard transfemoral access. We report the case of a 65-year-old woman with a history of recurrent deep vein thrombosis and a permanent Greenfield IVC filter implanted approximately 15 years prior, who presented with symptomatic paroxysmal atrial fibrillation and intolerance to multiple antiarrhythmic medications. Pulmonary vein isolation was successfully performed via a femoral approach, with guidewires and catheters carefully advanced through the peripheral struts of the filter under fluoroscopic and transesophageal echocardiographic guidance. The procedure was completed without complications. At the sixth-month follow-up, including Holter monitoring, the patient remained in sinus rhythm without recurrence. This case highlights the feasibility of transfemoral pulmonary vein isolation in selected patients with an IVC filter using careful technique and imaging guidance.

## Introduction

Atrial fibrillation is a common arrhythmia frequently requiring rhythm control strategies such as catheter ablation in symptomatic patients [1]. Pulmonary vein isolation (PVI) is the cornerstone of interventional management, particularly in patients intolerant to antiarrhythmic drugs [1]. The standard PVI procedure is performed via transfemoral venous access through the inferior vena cava (IVC) [2]. However, the presence of an IVC filter may hinder catheter navigation, increase procedural complexity, and expose patients to potential complications such as filter displacement or guidewire entrapment [2]. Currently, no specific guidelines addressing catheter ablation in patients with IVC filters, and management remains individualized. We report a case of successful transfemoral PVI performed through a permanent IVC filter, highlighting technical considerations and procedural feasibility.

## Case presentation

A 65-year-old woman presented with exertional dyspnea and intermittent blurred vision. Her medical history was notable for recurrent deep vein thrombosis requiring long-term anticoagulation and placement of a permanent Greenfield IVC filter approximately 15 years prior.

**Figure 1:**
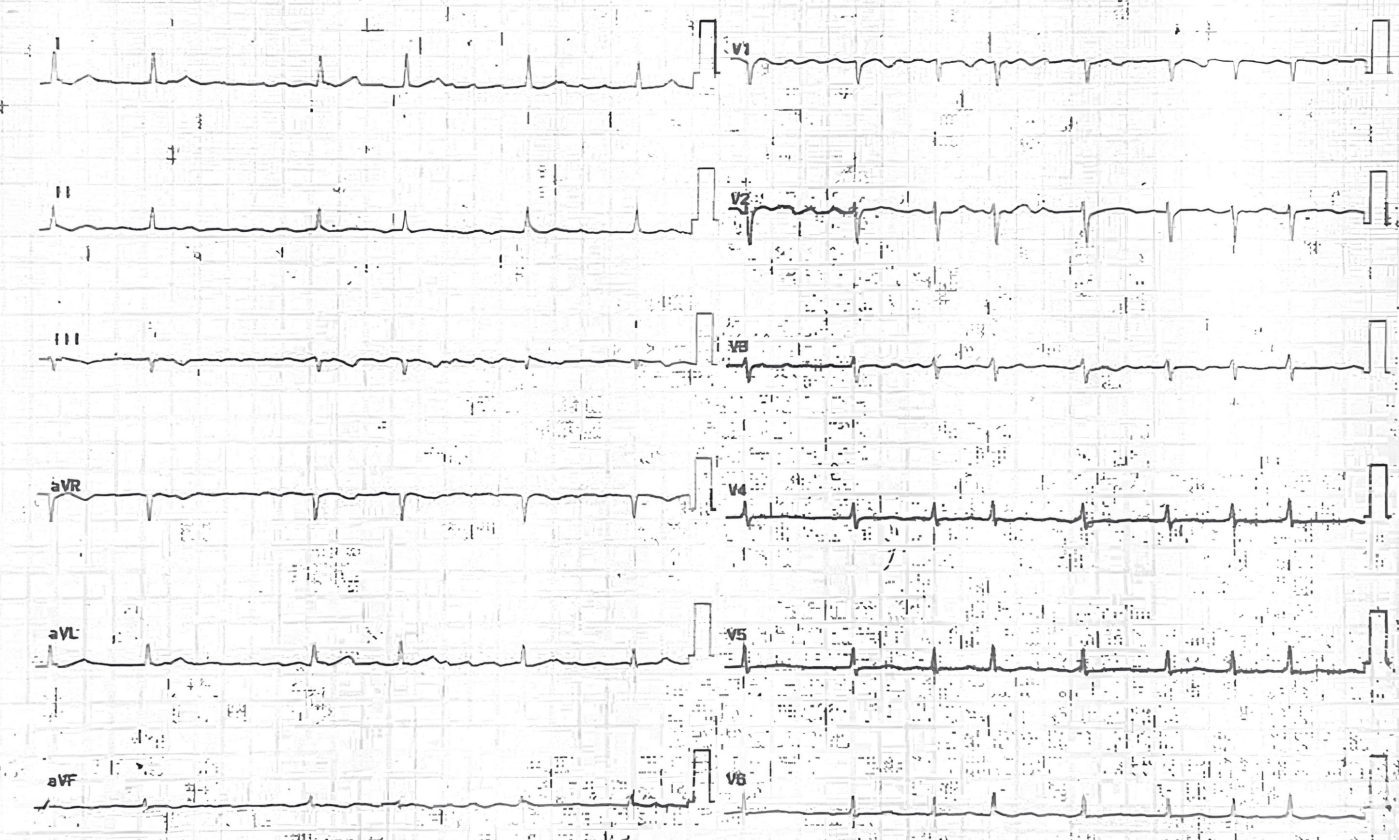
Initial electrocardiogram revealing atrial fibrillation.

On clinical examination, the patient was hemodynamically stable with a blood pressure of 110/70 mmHg and a heart rate of 115 beats/minute. There were no signs of peripheral congestion, and cardiac auscultation was unremarkable. An electrocardiogram (EKG) revealed atrial fibrillation with a rapid ventricular response at 115 beats/minute (Figure 1).

**Figure 2:**
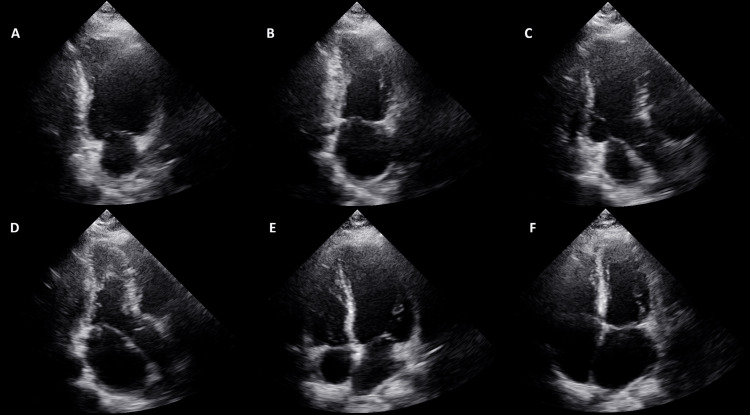
Initial transthoracic echocardiography demonstrating preserved left ventricular systolic function and no structural abnormalities. (A) Apical two-chamber view in diastole. (B) Apical two-chamber view in systole. (C) Apical three-chamber view in diastole. (D) Apical three-chamber view in systole. (E) Apical four-chamber view in diastole. (F) Apical four-chamber view in systole.

Transthoracic echocardiography showed preserved left ventricular function and no structural abnormalities (Figure 2). Initial pharmacologic management included bisoprolol 1.25 mg, which was discontinued due to poor tolerance, and the patient was switched to flecainide, which restored sinus rhythm on follow-up EKG.

**Figure 3:**
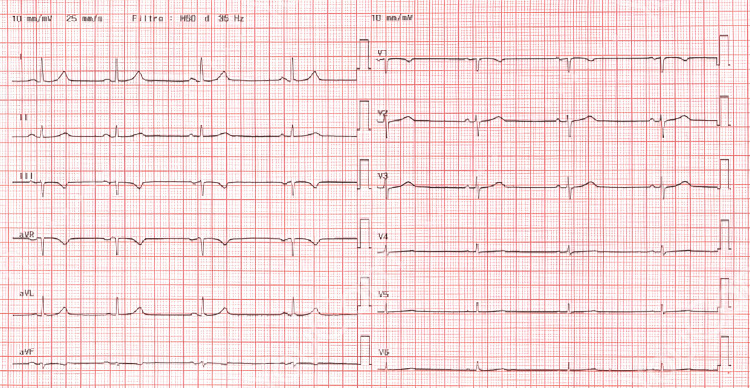
Follow-up electrocardiogram revealing sinus bradycardia.

Two weeks later, the patient reported polyuria along with visual disturbances, significant fatigue, and dizziness, consistent with intolerance to flecainide, prompting a switch to sotalol. However, during subsequent follow-up, she developed symptoms suggestive of bradyarrhythmia. EKG revealed sinus bradycardia at 43 beats/minute, with a PR interval of 158 ms, normal QRS duration and axis, and a corrected QT interval (QTc) of 426 ms (Figure 3). Additional laboratory testing identified previously undiagnosed hypothyroidism, which was promptly treated with levothyroxine. Despite normalization of thyroid function, the patient continued to exhibit bradyarrhythmia under antiarrhythmic therapy, and Holter monitoring confirmed recurrent paroxysmal atrial fibrillation, supporting the indication for catheter ablation.

Given her symptomatic paroxysmal atrial fibrillation and intolerance to multiple antiarrhythmic drugs, the patient was referred for catheter ablation with PVI. The presence of an IVC filter raised concerns about vascular access and catheter navigation. A preprocedural venogram was performed to confirm filter patency and exclude thrombus. The procedural strategy consisted of advancing guidewires through the peripheral struts while avoiding the central portion of the filter.

**Figure 4:**
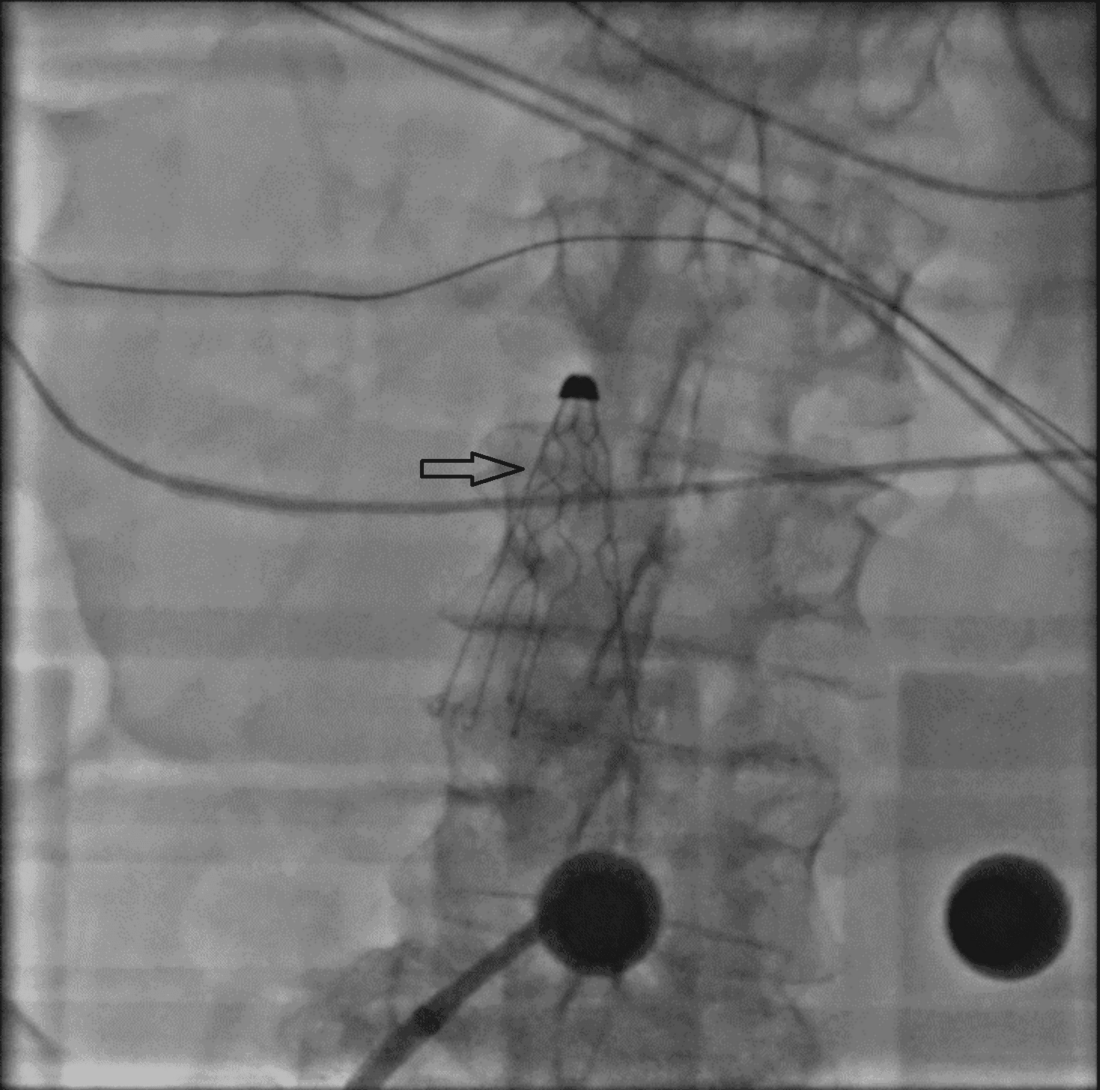
Fluoroscopic image demonstrating the patency of the inferior vena cava filter.

The ablation was performed under general endotracheal anesthesia, with transesophageal echocardiography (TEE) used intermittently during transseptal puncture and post-ablation assessment. As part of the preprocedural planning, venography was performed to confirm the position, patency, and integrity of the permanent Greenfield IVC filter and to exclude thrombus formation (Figure 4). Given the absence of thrombus and preserved IVC flow on venography, additional CT imaging was deemed unnecessary to avoid unnecessary contrast exposure and radiation.

Fluoroscopic imaging ensured that guidewires introduced via the femoral vein approached the IVC filter through the peripheral struts without traversing the central hub. A 10-Fr sheath was inserted into the right femoral vein. Femoral arterial access was limited to a 4-Fr catheter for pressure monitoring, without arterial dilation. The procedure was performed under anticoagulation with intravenous unfractionated heparin, administered to maintain an activated clotting time between 300 and 350 seconds. A 0.032-inch J-tipped guidewire was carefully advanced through the peripheral struts of the filter under fluoroscopic guidance, followed by the introduction of a 7-Fr GL catheter (Medtronic AVE), which crossed the filter smoothly without resistance or deformation of the device. Hemodynamic assessment demonstrated normal filling pressures and right ventricular pressures.

**Figure 5:**
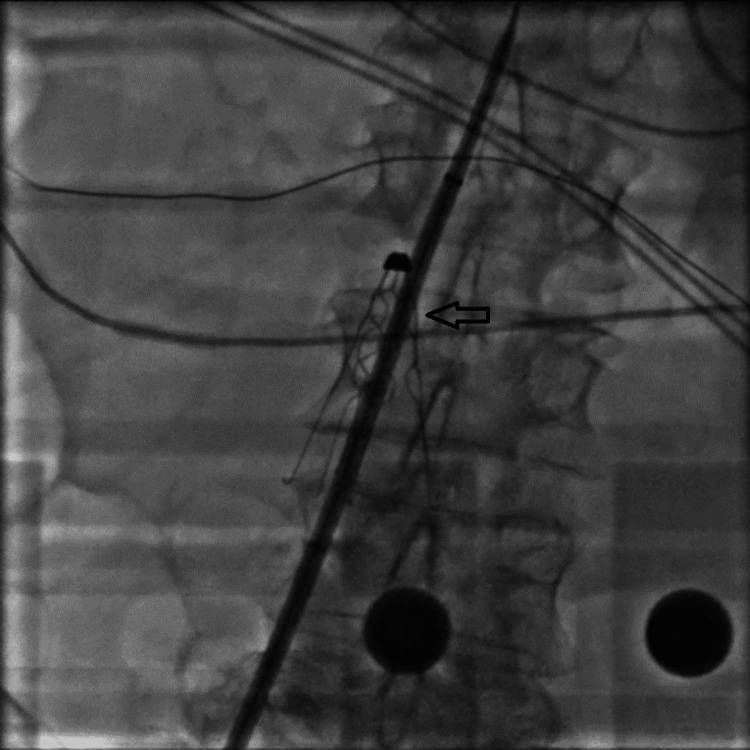
Fluoroscopic view (anteroposterior projection) showing guidewire and catheter passage through the peripheral struts of the inferior vena cava filter.

**Figure 6:**
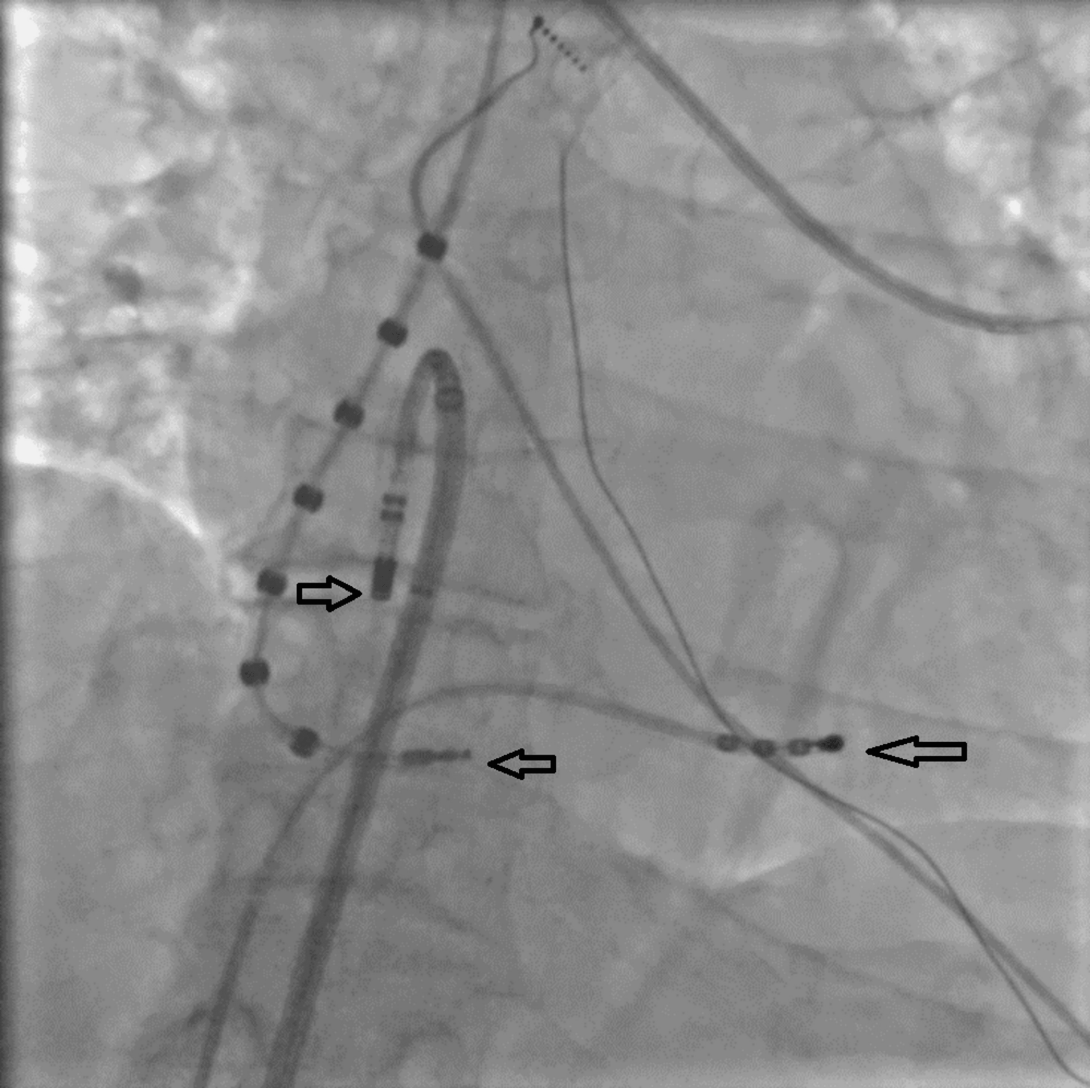
Fluoroscopic image (anteroposterior projection) showing catheter positions during the procedure, including a coronary sinus catheter and an esophageal temperature probe.

An SL0 transseptal sheath (St. Jude Medical) was advanced over the guidewire, and transseptal puncture was performed under combined fluoroscopic and TEE guidance using a standard Brockenbrough needle. Through this sheath, a circular mapping catheter and an irrigated contact force-sensing ablation catheter (SmartTouch™, Biosense Webster) were sequentially advanced into the left atrium. Three-dimensional (3D) electroanatomical mapping and navigation were performed using the CARTO system (Biosense Webster), allowing precise localization of the pulmonary vein ostia and accurate lesion delivery. All catheter manipulations across the IVC filter were performed cautiously to prevent displacement or structural compromise of the device (Figures 5, 6).

**Figure 7:**
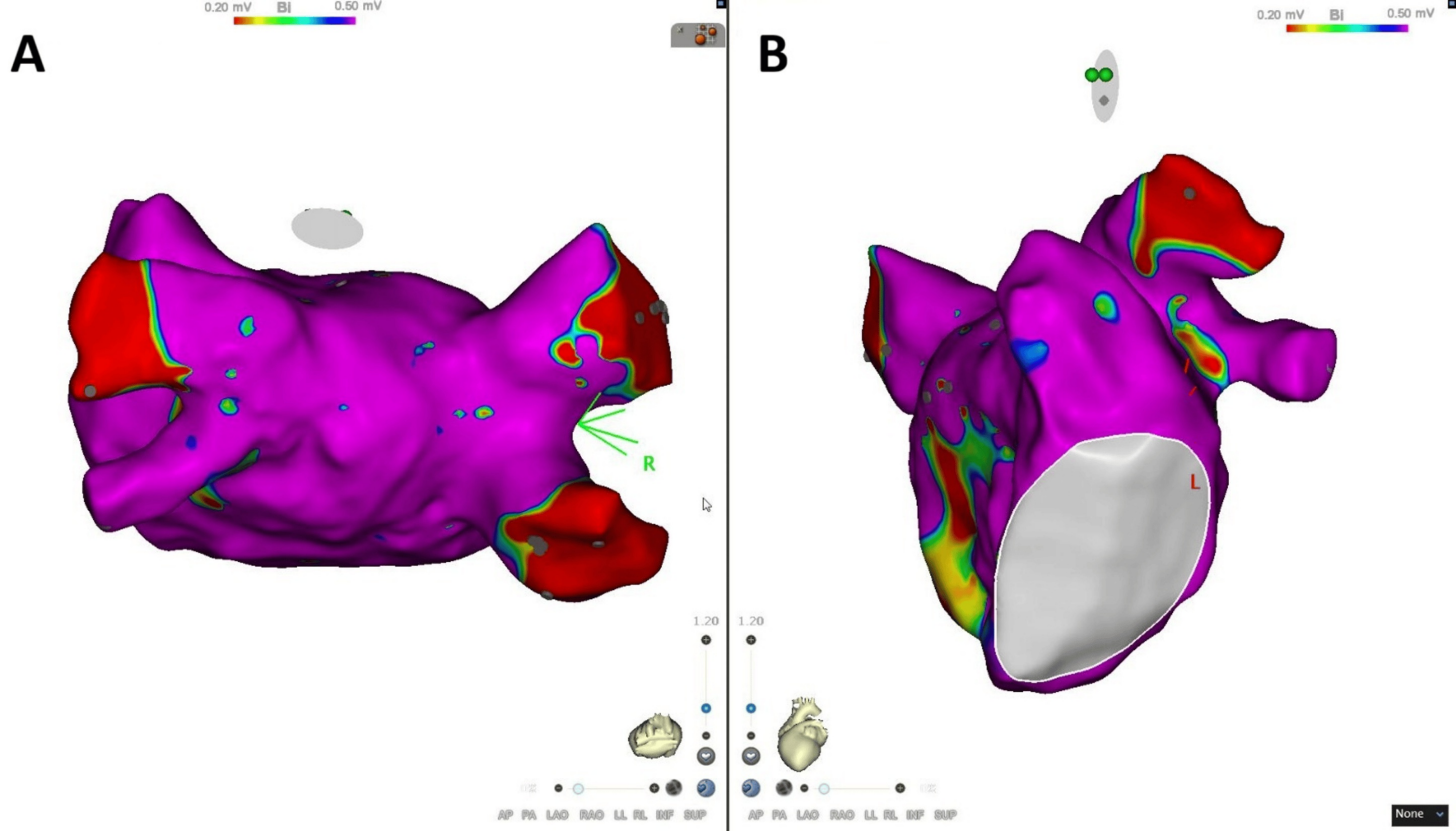
Pre-ablation three-dimensional electroanatomical voltage map of the left atrium showing pulmonary vein ostia. (A) Superior view. (B) Lateral view.

Using a 3D electroanatomical mapping system (CARTO, Biosense Webster), a pre-ablation voltage map of the left atrium was obtained to localize the pulmonary vein ostia and assess conduction (Figure 7).

**Figure 8:**
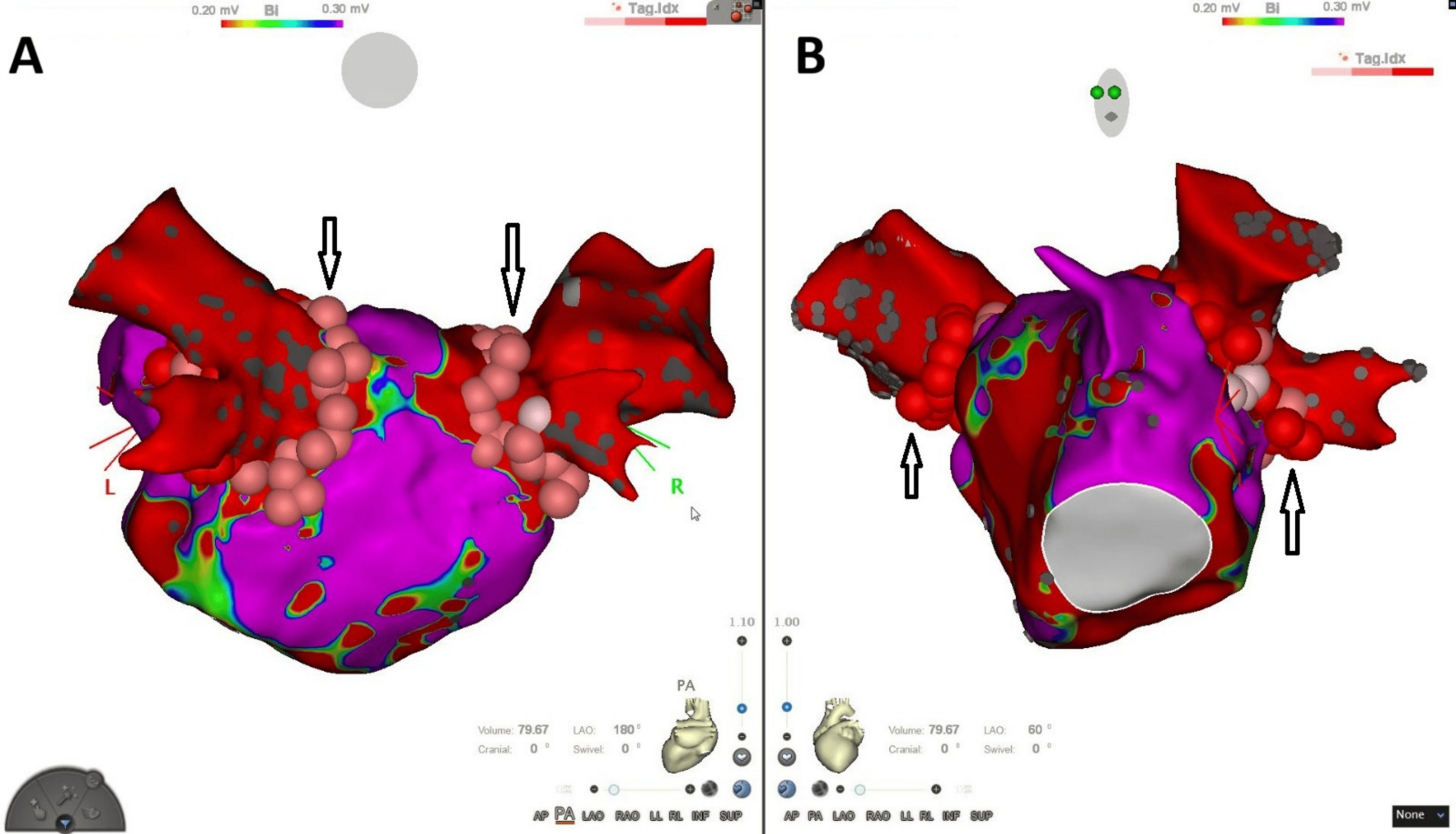
Post-ablation three-dimensional electroanatomical voltage map confirming complete pulmonary vein isolation. (A) Posterior view. (B) Lateral view.

The procedure was completed successfully without complications, and all catheters were safely withdrawn through the filter without resistance or evidence of device deformation. A post-ablation voltage map confirmed a complete entrance block in all four pulmonary veins (Figure 8).

Final fluoroscopic and TEE evaluation confirmed that the IVC filter remained intact and correctly positioned, with no evidence of deformation or thrombus formation. Oral anticoagulation with apixaban 5 mg twice daily was continued after the procedure. During a six-month follow-up period, the patient remained asymptomatic, with no recurrence of atrial fibrillation on outpatient evaluation or ambulatory rhythm monitoring, including Holter recordings.

## Discussion

According to the European Society of Cardiology guidelines, IVC filters are recommended in patients with acute pulmonary embolism who have an absolute contraindication to anticoagulation, or in those with recurrent pulmonary embolism despite adequate anticoagulation [3]. However, the indications for IVC filter use have significantly expanded in recent years, extending beyond established guidelines [4]. These include cases of extensive clot burden, impaired cardiopulmonary reserve, preparation for thrombolysis, and prophylactic use in high-risk patients [4]. Although many of these emerging applications lack strong evidence, these trends highlight the need for further scientific evaluation to better define the risk-benefit profile of IVC filters in such contexts, especially considering their potential for complications [4].

PVI is typically performed through the IVC via femoral vein access [2]. This technique involves the use of two or three long, preshaped sheaths to facilitate catheter insertion and positioning [2]. Following sheath placement, an atrial-septal puncture is performed to access the left atrium, often guided by fluoroscopy and echocardiography [2]. This approach allows for precise navigation and positioning of the ablation catheter at the pulmonary vein ostia, where energy is delivered to achieve isolation and disrupt the arrhythmic triggers [2].

However, the presence of an IVC filter represents a technical challenge for transfemoral procedures, as it may hinder catheter navigation and stability [5]. This is particularly relevant during PVI, where precise catheter control is essential [5]. The presence of an IVC filter may impede guidewire and catheter passage, increasing procedural complexity and the risk of complications [2]. Reported adverse events include filter migration or displacement, venous perforation, pulmonary embolism, and guidewire entrapment [2].

When transfemoral access is not feasible, alternative approaches include surgical ablation, retrograde aortic access, transhepatic access, or superior venous access via the jugular or subclavian veins [6,7]. However, these strategies are generally more invasive or technically challenging and may be associated with increased procedural risk [6,7].

Despite these challenges, emerging evidence supports the feasibility and safety of transfemoral access in the presence of an IVC filter [5]. Previous reports have demonstrated that careful catheter and sheath manipulation across the filter can be performed safely in selected patients, particularly when guided by fluoroscopy and appropriate procedural strategies [5]. For example, Kanjwal et al. reported successful advancement of multiple catheters and sheaths across an IVC filter without major complications, emphasizing the importance of avoiding the central filter struts and using careful fluoroscopic guidance to facilitate safe navigation [5].

Similarly, Masaki et al. described a reproducible strategy for PVI in patients with an IVC filter, combining modified transfemoral access with adjunctive imaging techniques to enhance procedural safety and catheter control [2]. Their approach also highlighted the value of detailed preprocedural assessment to guide catheter navigation and optimize procedural outcomes [2].

This case illustrates the feasibility of performing PVI via a transfemoral approach in the presence of a permanent IVC filter, using careful catheter navigation through the peripheral struts under fluoroscopic and echocardiographic guidance. Beyond confirming feasibility, it provides practical procedural insight by demonstrating that contemporary techniques, including 3D electroanatomical mapping and contact force-guided ablation, can be safely applied in this challenging anatomical setting.

## Conclusions

This case demonstrates the feasibility of performing PVI via a transfemoral approach in the presence of a permanent IVC filter, using careful catheter navigation through the peripheral struts under fluoroscopic and echocardiographic guidance. It highlights that contemporary techniques, including 3D electroanatomical mapping and contact force-guided ablation, can be safely applied in this setting. However, given the single-case design and limited follow-up, further studies are needed to confirm the safety and reproducibility of this approach.
